# Strategy for Constructing Phosphorus-Based Flame-Retarded Polyurethane Elastomers for Advanced Performance in Long-Term

**DOI:** 10.3390/polym15183711

**Published:** 2023-09-08

**Authors:** Yuxin Luo, Zhishuai Geng, Wenchao Zhang, Jiyu He, Rongjie Yang

**Affiliations:** National Engineering Technology Research Center of Flame Retardant Materials, School of Materials Science & Engineering, Beijing Institute of Technology, Beijing 100081, China

**Keywords:** polyurethane elastomer, phosphorus flame retardant, reactive flame retardant, fire protection

## Abstract

Polyurethane elastomer (PUE), which is widely used in coatings for construction, transportation, electronics, aerospace, and other fields, has excellent physical properties. However, polyurethane elastomers are flammable, which limits their daily use, so the flame retardancy of polyurethane elastomers is very important. Reactive flame retardants have the advantages of little influence on the physical properties of polymers and low tendency to migrate out. Due to the remarkable needs of non-halogenated flame retardants, phosphorus flame retardant has gradually stood out as the main alternative. In this review, we focus on the fire safety of PUE and provide a detailed overview of the current molecular design and mechanisms of reactive phosphorus-containing, as well as P-N synergistic, flame retardants in PUE. From the structural characteristics, several basic aspects of PUE are overviewed, including thermal performance, combustion performance, and mechanical properties. In addition, the perspectives on the future advancement of phosphorus-containing flame-retarded polyurethane elastomers (PUE) are also discussed. Based on the past research, this study provides prospects for the application of flame-retarded PUE in the fields of self-healing materials, bio-based materials, wearable electronic devices, and solid-state electrolytes.

## 1. Introduction to Polyurethane Elastomers

**Figure 1:**
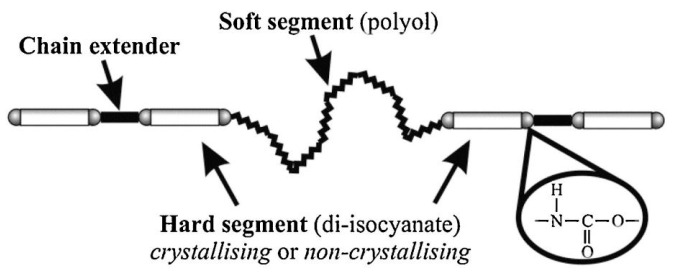
Micro-phase separation of polyurethane [6]. Copyright 2019, Elsevier.

Polyurethane elastomer (PUE) is a typical block polymer with alternately arranges soft and hard segments [1,2]. Isocyanates and small molecular chain extenders constitute the hard segment, and the soft segment is composed of polyols (shown in Figure 1). Compounds containing two or more -OH groups with a molecular weight of more than 500 g/mol are defined as polyols, while those smaller molecules with hydroxyl groups and/or amines are regarded as chain extenders.

The abundant hydrogen bonds physically cross-link the linear chains to form network structures, resulting in microscopic phase separation between soft and hard segments [3]. The mobility of the soft segment makes the PUE elastic, while the hard segment hinders the rotation of the molecular chain, giving the PUE hardness and mechanical strength [4]. The unique micro-phase structure of PUE endows it with wear resistance, toughness, and good processability of thermoplastics [5]. The combination of “tough and strong” greatly expands the application of PUE.

The soft segment offers the flexibility of PUE chains. The polyols used to synthesize PUE can be classified into polyether polyols, polyester polyols, and polyolefin polyols. Polyester polyols can be further divided into aliphatic polyester polyols and aromatic polyester polyols [7]. Polyolefin polyols are polyols containing -C=C- bonds in the repeating units, including polybutadiene polyols, polybutadiene acrylonitrile polyols, and the like. The comparisons of various types of polyols are summarized in the table below (Table 1).

The hard segment of polyurethane elastomer is usually composed of isocyanate and chain extender, and the additional reaction of alcohol (-OH) to isocyanate (-NCO) is the basis for synthesizing polyurethane. PUE is generally synthesized with di-functional isocyanates, shown in Table 2 [9,10]. Aromatic isocyanates, such as toluene di-isocyanate (TDI) and methylene diphenyl di-isocyanate (MDI), have high reactivity and low production costs, and they are usually used to manufacture rigid products. However, products made from aromatic isocyanates tend to turn yellow over time due to their photo-oxidative instability. In contrast, products based on aliphatic di-isocyanates have excellent hydrolysis resistance and yellowing resistance in spite of their low reactivity and comparatively high expense. The common aliphatic isocyanates include hexamethylene di-isocyanate (HDI), isophorone di-isocyanate (IPDI), and 4,4′-diisocyanate dicyclohexylmethane (HMDI), which is a hydrogenation product of MDI [8].

For most polyurethanes (poly(urea-urethanes)), soft segments and isocyanates account for more than 95% of the mass, and they are linked by lower molecular weight diols or di-amines chain extenders. The most commonly used chain extenders include 1,4-butanediol (BDO), ethylene glycol, and so on.

There are many choices of soft segments and hard segments for synthetic polyurethane elastomers, so adjusting the type and proportion of starting materials offers polyurethane elastomer a wide range of properties, synthesis routes, and processing methods. Contemporarily, coating materials based on polyurethane elastomers are widely used in various fields, such as construction, transportation, electronics, and aerospace, because of their good wear resistance, oil resistance, high elasticity, and high transparency [8,11,12]. However, the thermo-stability of polyurethane elastomer is not as desirable, and it has been defined as a flammable polymer [13]. A large number of toxic fumes causing poisoning and suffocation, such as CO, HCN, NO_x_, and –NCO group-containing compounds, are produced in the combustion of PUE [14,15,16], which causes great harm to human health and property safety. In 2021, a total of 748,000 fires were reported in China, with more than 4000 casualties and direct property losses of CNY 6.75 billion. The frequent occurrence of fires drives the attention of various industries to the flame retardancy of PUE materials [17,18,19]. To keep a balance of environmental sustainability and rapid development, various countries have successively put forward flame-retardant standards for polyurethane, and it becomes essential to develop novel flame-retarding methods for polyurethane elastomers with great efficiency and less adverse environmental effects.

## 2. Research the Status of Reactive Flame-Retarded Polyurethane Elastomer

**Figure 2:**
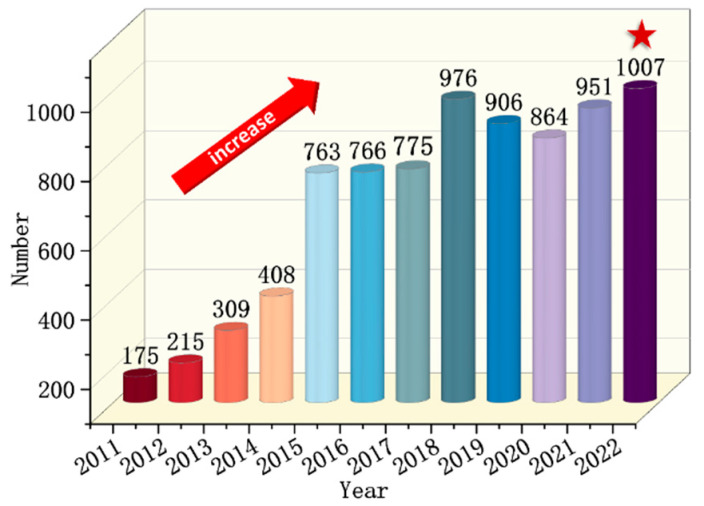
Research progress reflected by the number of published papers on flame-retarded polyurethane (excluding foam) in the past ten years [4,20,21,22].

In order to provide a more comprehensive understanding of the research status of the flame retardancy of polyurethane elastomer, we analyzed relevant literature concerning flame retardancy of polyurethanes (foams, another extensively reported non-elastomer polyurethane materials, were excluded to keep the focus of the analysis on polyurethane coating materials) over the past two decades by using the formula ((TS = polyurethane) and (TS = flame retard* OR TS = fire retard*) NOT (TS = foam)) in the advanced search of web of science, including reviews, articles, and conferences. Figure 2 indicates that the number of research papers on flame-retardant polyurethane has gradually increased in recent years, especially after 2014. As of 30 July, 428 papers on flame-retarded polyurethane (except foam) were already published in 2023.

Flame retardants used in polyurethanes are usually divided into two types: additive flame retardant and reactive flame retardant. Additive flame retardant is physically incorporated into polyurethane elastomers [23,24,25]. Due to its convenient usage and low cost, additive flame retardant still dominates the flame-retardant market [26,27,28]. However, additive flame retardants have many disadvantages: (1) High dosage addition results in the decline in mechanical performance of PUEs. Finding the appropriate additive ratio to balance the flame-retarding efficiency and mechanical properties is a constant goal in development of contemporary additive flame retardants; (2) additive flame retardants tend to migrate out from the materials over time, and the problems of persistence, bioaccumulation, and toxicity (PBT) remain to be solved [29]. Macromolecular/oligomeric flame retardants are under development as novel and effective solutions in recent years. For example, a phosphorus–piperazine-containing additive was designed to fabricate flame-retarding high-performance thermoplastics polyurethane (TPU) by Chen et al. [30], which improved flame retardancy and reduced the amount of additives.

The reactive flame retardant refers to the flame-retardant elements or structural units incorporated into the polyurethane elastomer via covalent bonding. It has been proven to address the above problems effectively, especially reducing the tendency to migrate out, leading to long-term flame retardancy. There are two general methods of preparing reactive flame-retarded polyurethanes: to introduce flame-retardant elements/structural units into chain extenders and to graft flame-retarding structural units into soft segments (polyols) [20]. PUE can be either thermoplastic or thermoset, so the flame-retardant chain extenders can also function as crosslinkers in thermoset PUE. For example, many bio-based compounds rich in phenol rings are often employed as cross-linkers while efficiently inhibiting flame spreading [31,32].

With the increase in flame-retarding requirements in various industries, intrinsic flame-retarded (reactive flame retardant) polyurethane receives more attention because of its characteristics of long-term flame retardancy and environmental friendliness. However, compared with additive flame retardant, the preparation of reactive flame retardant is more challenging, leading to fewer related studies. Once facile and efficient methods for synthesizing reactive flame retardants are established, they will become research hotspots with broad development prospects.

### 2.1. From Halogen-Containing Flame Retardant to Halogen-Free Flame Retardant

Halogen-containing flame retardants have high flame-retarding efficiency, and they were widely used in the last century. However, corrosive gases are produced while the flame is inhibited and cause great harm to the environment [33]. With the proposal of sustainable development and green flame retardancy, halogen-containing flame retardants are withdrawing from the historical stage of flame retardant [4].

Meanwhile, flame retardants containing phosphorus, nitrogen, and silicon have played an increasingly important role in replacing traditional halogen-containing flame retardants due to their excellent performances. According to the statistics, P-containing flame retardants own the highest number of related reports, so it can be concluded that P-containing flame retardants are the most promising substituents for halogen-free flame retardants for polyurethanes.

**Figure 3:**
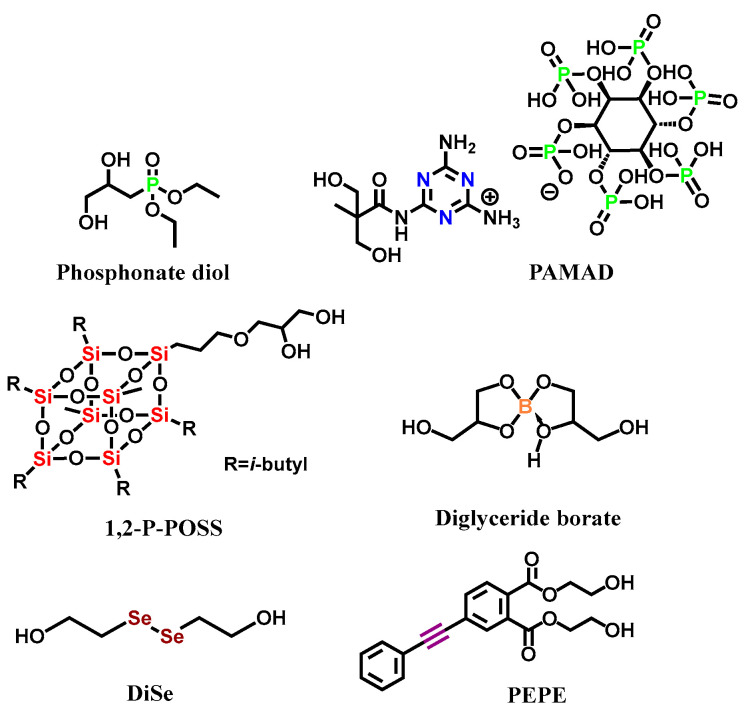
Examples of reactive flame retardants containing P, N, Si, B, and Se and thermal cross-linking flame retardant [34,35,36,37,38,39].

The following Figure 3 summarizes the structures of the representative P, N, Si, B, and Se reactive flame-retarding chain extenders, as well as the thermal crosslinking type flame-retarding chain extenders, which are not based on specific flame retarding elements. Section 2.2, Section 2.3, Section 2.4, Section 2.5, Section 2.6 and Section 2.7 will briefly introduce the flame-retarding effect and the corresponding mechanism to provide a basic grasp of each type.

### 2.2. P-Containing Reactive Flame Retardant

Phosphorus-based flame retardants (P-FRs) are considered one of the most effective substitutes for halogen-containing flame retardants [40]. The phosphorus-based flame retardants function through two mechanisms, including (1) producing radicals in the gas phase to inhibit the flame and (2) facilitating the carbonization in the condensed phase to insulate the combustibles from the flame above. Section 3.2 will analyze the flame-retardant mechanisms of P-FRs in detail. For example, B. Mortaigne et al. [15] synthesized a novel chain extender and applied it in polyurethane (1 in Figure 3). Thermo gravimetric analysis (TGA) showed that a phosphorous-containing protective layer formed on the polymer surface and effectively enhanced thermal stability. The char formation was promoted while PO• was generated to quench the combustion radicals, which efficiently stopped the fire [22,41]. The following Section 4 will systematically sum up more cases of the design and application of P-containing reactive flame-retarded polyurethane.

### 2.3. N-Containing Reactive Flame Retardant

Nitrogen-containing reactive flame retardants are generally realized through grafting N-containing functional units, such as melamine groups, into the polyurethane structure via covalent bonds [42]. In addition to melamine, triazole, tetrazole, and imidazole have gained increasing attention (due to their more facile synthesis) for diluting the flammable gas concentration and forming a more stable carbon layer, such as graphitized carbon nitride (g-C_3_N_4_). Gu et al. [35] synthesized the “Three sources in one” intumescent chain extender PAMAD and the corresponding polyurethane(PWPU). With the increase in PAMAD content, the peak heat release rate (PHRR) and total heat release (THR) of PWPU reduced by 51.2% and 33.8% and reached UL-94 V-0 level. Nitrogen-containing flame retardants may require synergistic interactions with other additives or compounds to achieve optimal performance. Thus, there were, comparatively, few reports on the application of N-containing reactive flame retardant in polyurethane, alone, and P-N synergistic flame retardant was more widely employed due to its excellent efficiency. Section 5 will focus on the classification and application of P-N synergistic flame retarded polyurethane in recent years.

### 2.4. Silicon-Containing Reactive Flame Retardant

Silicon-based flame retardants are usually composed of stable Si-O-Si bonds, with characteristics of low toxicity, anti-drip, easy carbonization, and great smoke suppression. The representative of Si-containing flame retardant is POSS (Polyhedral oligomeric silsesquioxane). Nowadays, POSS has been widely used in various polymeric materials [43,44,45,46,47,48]. Kwon et al. [36] used reactive POSS diols as chain extenders to synthesize flame-retarded PU(FRPU) by the one-step method. Their work indicated that POSS promoted the formation of dense carbon layers, embedded with nano-sized SiO_2_ droplets, during combustion to increase the compactness and robustness. Silicon-based flame retardants may have compatibility issues with certain polymers or additives commonly used in formulations, impacting the overall performance of the flame-retardant system.

### 2.5. Boron-Containing Reactive Flame Retardant

Boron-containing flame retardants (B-FRs) have also become popular because of their low smoke, non-toxicity, and excellent flame retardancy. Liu [37] et al. synthesized a boron-containing polyurethane. It was indicated that, with the increase in boron content, the LOI of PU was increased to 30%, and the PHRR and THR decreased by 27.6% and 38.15%, respectively. As with silicon-containing flame retardant, boron-containing flame retardant acts through a condensed phase flame-retardant mechanism, and it forms dense boron-carbon layers to delay or terminate the transfer of heat. The cost and availability of boron-containing flame retardants could be limiting factors. Some B-FRs were expensive, making them less practical for widespread use, especially in cost-sensitive applications.

### 2.6. Selenium-Containing Reactive Flame Retardant

Selenium is used infrequently in the construction of polyurethanes, although this element has drawn significant attentions in the field of dynamic polymers/self-healing polymers due to its sensitivity to light stimulus, making Se-Se-containing elastomers unique types of materials. On the other hand, selenium quantum dots have been reported to be able to scavenge active radicals at a modest condition, demonstrating the potential to reduce flame risk by consuming oxygen (mainly the gas phase mechanism) [39]. Jin et al. [39] reported the first example of applying organo-selenium-containing reactive flame-retarded TPU. Both high self-healing efficiency (91.25% after 30 min treatment in photoreactor) and favorable flame retardancy (LOI: 28.5%; UL-94: V-0; self-extinguishing time: <1 s; THR: 49.28 MJ/m^2^) were achieved at the same time, boding well for this novel class of high-performance polymer design. However, selenium-containing flame retardants were unstable, and there were few reports regarding them at present.

### 2.7. Thermal Cross-Linking Reactive Flame Retardant

In recent years, the flame-retarded PU without the incorporation of traditional flame-retardant elements in its molecular backbone have been reported [49,50,51,52]. For instance, thermal-induced crosslinking groups, such as alkynyl, benzene ring, and carbon-nitrogen double bond, were introduced into the polymer structure and demonstrated unique flame-retarding behavior. He et al. [38] successfully synthesized an aromatic acetylene derivative PEPE, which was used as the chain extender to partially replace BDO. The arylalkynyl structure formed a cross-linked network and foaming char structure after burning. The PHRR and THR decreased by 46.2% and 24.5%, respectively, and the tensile strength of TPU, containing 1.35 wt. % PEPE, was 39.2 MPa, which was comparable and even higher than non-flame-retarded TPU. Wang et al. [53] synthesized oligomers containing phosphorus and alkyne groups simultaneously, and they used them as additive flame retardants to achieve the anti-dripping performance of TPU, demonstrating the huge potential of this new type of nontraditional flame retardants [30,53]. However, thermal cross-linking reactive flame retardant is still at an early stage and requires more systematic investigations.

Above all, the developments of reactive flame retardants are essential to simplifying the processing methods of PUE while keeping its high mechanical performance and flame-retarded properties. Phosphorus, a versatile and effective element used in flame retardants, has the best potential to become a halogen-free flame retardant alternative so far. The following sections will discuss the application of phosphorus-containing flame retardants and the corresponding flame-retarded polyurethane elastomers in more details.

## 3. Flame Retarding Mechanism of P-FRs in PUE

### 3.1. Thermal Decomposition Behavior of P-FR Polyurethane

Once the polyurethane is ignited, toxic gases are produced with significant life hazards, so flame retardancy and thermal stability of PU are extremely important. Thermal decomposition of PU is divided into at least two stages [15,54]. Firstly, carbamate bonds are cleaved to transform polyurethanes into oligomers and small molecules, such as isocyanates, alcohols (amines), and carbon dioxide. When the temperature continues to rise, the soft segments (polyols) begin to decompose, while diffusing into the flame area, and form flammable blends with oxygen in the air to promote combustion.

Generally, with the addition of P-FRs, the thermal decomposition temperature and thermal stability decrease, with a few exceptions documented in recent reports [55,56]. Thus, whether the thermal stability of flame retardants matches that of the matrix is an important consideration. Alleviation or prevention of matrix degradation will only be effectively achieved if the flame retardant decomposes first.

### 3.2. General Flame Retarding Mechanisms of P-FRs

**Figure 4:**
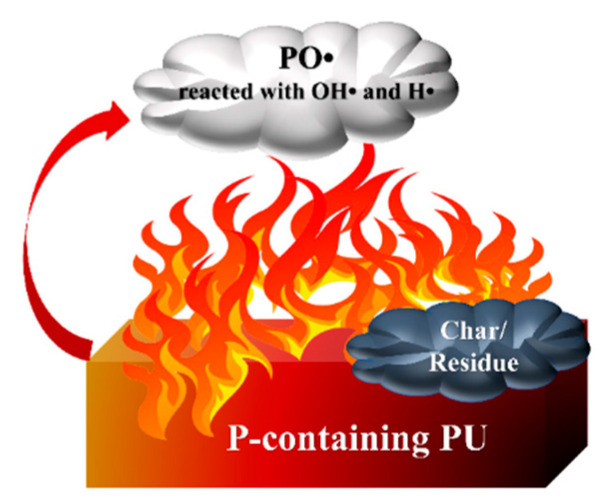
Gas and condensed phase flame-retardant mechanisms of P-FRs.

Most flame retardants exhibit two flame-retardant mechanisms, including the gas phase and the condensed phase (shown in Figure 4). Both of them effectively prevent the spread of flames and ensure the safety of people’s lives and properties.

In the condensed phase, phosphorus-containing compounds are turned into phosphoric acid and poly-phosphoric acid upon heating. Phosphoric acid has a strong dehydration ability, which promotes carbonization to form a closed carbon layer. Meanwhile, water, the other product of this transformation, absorbs heat and vaporizes, reducing the temperature around the material.

Meanwhile, the phosphorus-containing polymer releases incombustible gas during the decomposition process to dilute the concentration of combustible gas, which is usually defined as the gas-phase flame-retarding route. When the polymer is burned, a large number of radicals, such as H• and OH• radicals, are generated, which triggers a series of chain reactions and aggravates the combustion. Many P-containing flame retardants form PO•, which reacts with OH• and H• radicals, reducing their concentration and interrupting the combustion reaction. Most phosphorus-containing flame retardants have synergistic flame retardancy in both the condensed and gas phases [22].

### 3.3. Effect of Phosphorus Oxidation State on Flame Retarding Mechanism

**Figure 5:**
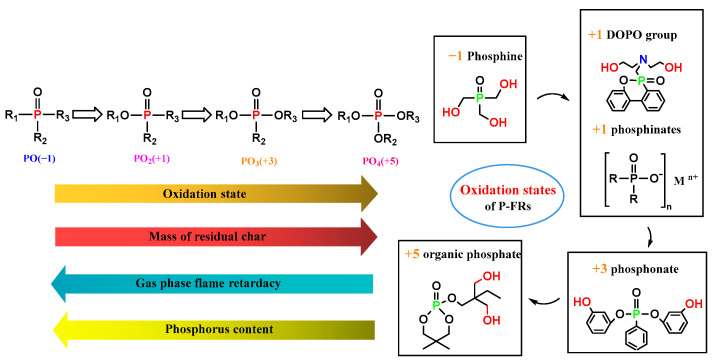
Different oxidation states impact flame-retardant behavior.

Phosphorus has different oxidation states (−1 to +5), which give rise to different flame-retardant mechanisms, as shown in Figure 5. When the substituting groups are basically the same (for example, R_1_ = R_2_ = R_3_), the phosphorus content (weight percent in the whole molecule) decreases while the oxidation state increases. Modesti et al. [57] investigated the effect of the P oxidation state on its FR behavior in polyurethane foams, finding that the amount of stable residue after burning increased, together, with less release of volatiles as the oxidation state of P increased. Therefore, the low oxidation state generally led to the gas-phase FR mechanism, while the high oxidation state facilitated the condensed phase FR mechanism [58,59]. These insights have significant impacts on the design of flame retardancy for polymer materials based on phosphorous.

## 4. Inherent Phosphorus-Containing Flame Retardants Applied in PUE

The state of the art of development, for the phosphorus-containing flame retardation of PUE, is going to be analyzed in depth in this part. Here, we focus on two kinds of inherent FRs (chain extenders and polyols), which constitute polyurethanes differently.

### 4.1. Application of Phosphorus-Containing Chain Extender in Flame-Retarded PUE

**Figure 6:**
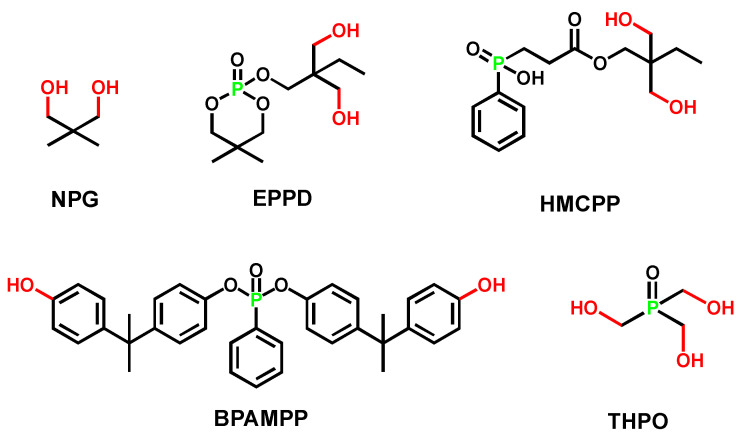
Chemical structure of typical P-containing chain extenders: BPAMPP, THPO, NPG, EPPD, and HMCPP [55,56,60,61].

Generally, adding P-FRs (whose structures were shown in Figure 6) decreases the thermal decomposition temperature of polyurethane. Table 3 showed that the T_5%_ of flame-retarded polyurethane decreased (PU chain extended with EPDD/HMCPP compared with NPG/BDO), which was due to the introduction of O-P=O groups (with decomposition temperature at 180 °C). The bond energy of the P-O bond and P=O bond were lower than that of the C-C bond, so the thermal decomposition was more likely to occur earlier [41].

Reactive FRs also affect the mechanical properties of TPU. Fan and Chiu [60,61] found that phosphoric acid/phosphate esters chain extenders could increase the elongation, at break, of polyurethane while deteriorating the tensile strength. The reason was that a small molecule chain extender was inserted in the polyurethane chains and weakened the inter-chain interactions, increasing mobility and reduction in crystallinity. Therefore, the plasticity of the polymer was increased, which was reflected by the increase in elongation at break.

As mentioned earlier, the temperature at 5 wt. % weight loss (T_5%_) would decrease with P-containing flame retardants incorporated into polyurethanes. However, the polyurethanes synthesized from BPAMPP or THPO are exceptions. Table 4 summarized the thermal stability and flame retardancy of polyurethanes with BPAMPP or THPO incorporated. The char residue, vertical combustion rating, and LOI exhibited an obvious improvement with the introduction of P-containing chain extenders, together, with a significantly lower PHRR and THR.

Huang et al. [55] reported a higher T_5%_ of polyurethane based on BPAMPP compared with the one with non-flame-retarded chain extender BDO. The increase in thermal stability was attributed to the rigid structure, containing two symmetric benzene rings, strengthening intermolecular force.

He et al. [56] reported another example of an increase in thermal stability of thermoset waterborne polyurethane elastomer. The introduction of tri-functional THPO was beneficial to the formation of a cross-linked protective layer, thereby preventing further thermal decomposition at high temperatures. THPO [56] followed the synergistic flame-retardant mechanisms of the gas phase and condensed phase. When the flame-retarded waterborne polyurethane (FWPU) was heated, phosphine oxide groups decomposed first, generating PO• radicals to capture H• and OH• radicals, interrupting the chain reaction related to combustion. Meanwhile, phosphoric acid layers aggregated on the polymer surface, and the carbonaceous char formed with a higher degree of graphitization linked by P-O-C and P-O-P bonds [62]. The dense char residues hindered the entry of oxygen and prevented heat transmission effectively, thereby enhancing the flame retardancy.

In order to further study the hydrolysis resistance of normal polyurethane (WPU) and flame-retarded polyurethane (PWPU-X), based on EPPD, Fan and co-workers [60] found that both the tensile strength and elongation at break decreased after hydrolysis, but they still remained above 90% of the original value. The reason was proposed to be that the phosphoester bonds formed a “core”, which was encapsulated by the “shells” of hydrophobic carbon cycles, preventing water from penetrating and attacking these hydrolysis-sensitive moieties so as to enhance the hydrolytic stability. Table 5 showed the excellent hydrolysis resistance of WPU, which facilitated industrial applications of this flame-retarded WPU.

### 4.2. Application of Phosphorus-Containing Polyols in Flame-Retarded PUE

**Figure 7:**
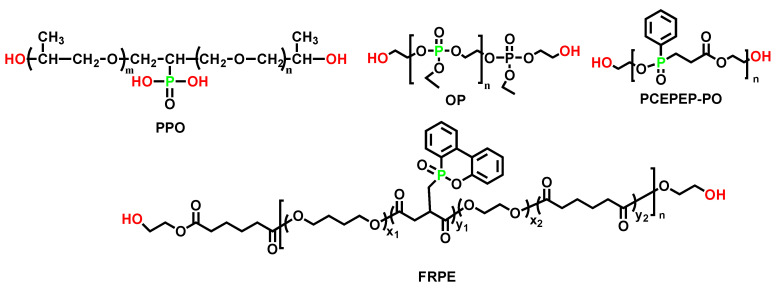
Chemical structure of P-containing polyols, including PPO, OP, P-FRPP, PCEPEP-PO, and FRPE [64,65,66,67].

Polyols are the major components of PUE, accounting for more than 60 wt. % of it. Flame-retarded polyols have been widely used in recent years because they can effectively prevent the migration of flame-retarded elements. The most famous P-containing polyols are Exolit^®^ OP 550 and OP 560 (with a similar structure to OP in Figure 7), which are used in the preparation of flame-retarded PU foam and waterborne PU coating dominantly [63]. This review aims to focus more on PUE based on flame-retarded Polyols.

Table 6 summarized four P-containing polyols and provided their chemical structures (Figure 7). With the increase in the content of phosphorus, LOI and char residue were improved, and the UL-94 V-0 rating was obtained. Li et al. [64] successfully synthesized a reactive phosphonate polyol (PCEPEP-PO). With 20 wt. % of PCEPEP-PO incorporated, the PUE achieved an LOI value of 28.8%, and THR was reduced by nearly 40%.

**Figure 8:**
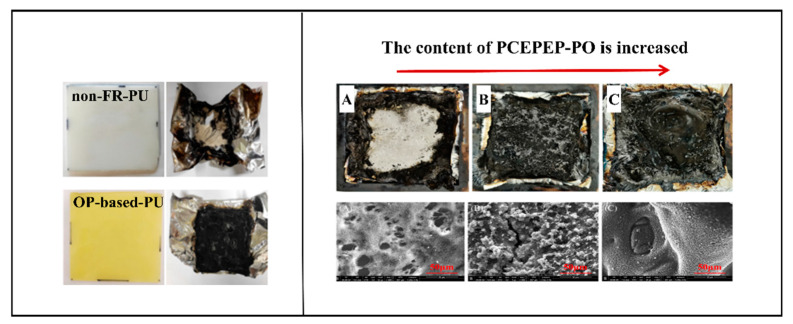
Cone calorimetry and SEM pictures of char residue of OP-based (**left**) and PCEPEP-PO-based (**right**) polyurethane (**A**–**C** represent PU and two types of flame-retarded PU with different phosphorous contents, respectively.) [65,66]. Copyright 2021, MDPI. Copyright 2021, John Wiley and Sons.

Flame-retarded PU could be prepared based on either polyether polyols, such as PPO, or polyester polyols, such as OP or PCEPEP-PO. Figure 8 showed the digital photographs of char residues of FR-PU after a cone calorimetry test. Thanks to the dehydrating effect of phosphate or phosphonate in each repeating unit, a dense carbon layer formed on the surface of the flame-retarded polyurethane, which protected the underlying polymer matrix from further burning and reduced the heat release. Scanning electron microscopy (SEM) provided more details of the char morphology after burning and indicated that, with the increase in P content in the flame-retarded PU, the carbon layer was denser and had fewer holes.

Mechanical properties of polyurethane were also strongly impacted by different types of P-containing polyols. Wang et al. [67] explored the relationship between the content of polyol, FRPE, and the mechanical properties of FR-PUE. With more FRPE incorporated, the tensile strength of FR-PUE increased from 16.3 Mpa to 25 Mpa, while the elongation at break decreased (Table 7A). This was explained by the enhanced phase separation of the soft segment and hard segment in FR-PUE, which contributed to the improvement of tensile strength. Meanwhile, increasing the content of the hard segment (28% to 37%) had a similar effect, and it was summarized in Table 7B. However, a further increase in the content of the hard segments (40%) resulted in a loss of toughness for the resultant FR-PUEs, which was a common trend observed in many reports.

Datta et al. [65] compared the TGA of flame-retarded polyurethane (FPU) based on OP in nitrogen and oxygen atmosphere (shown in Table 8). T_50%_ in oxygen and T_70%_ in both the oxygen and nitrogen of FPU were significantly higher than those of non-flame-retarded polyurethane, although T_5%_ of FPU, in both atmospheres, was significantly lower than non-flame-retarded REF. The early decomposition was likely to form stable oxidation products during the polyol degradation, preventing further decomposition of the rest of polyol. Thus, the flame-retarded polyurethane was demonstrated to be more resistant to overall degradation in oxygen-containing atmosphere, which made it more desirable in high-temperature fire scenarios.

Different hard segments also influenced the hardness and flame retardancy of PU based on the same P-containing polyol. Patel et al. [68] successfully synthesized a novel phosphorus-based polyol (tris-(*m*-hydroxy phenyl) phosphate), reacting with different di-isocyanates, to obtain a series of polyurethanes (EERPPU). The mechanical and flame-retardant properties are presented in Table 9, and it can be seen that IPDI and MDI-based systems give polyurethanes with higher molecular weight and thermal stability, implying that the degradation temperature and rate might be tightly related to the molecular weight. On the other hand, TDI and MDI-based polyurethanes (with LOI of 29% and 30%, respectively) offered higher flame retardancy because the aromatic ring structure was conducive to compact char formation in the combustion process.

**Figure 9:**
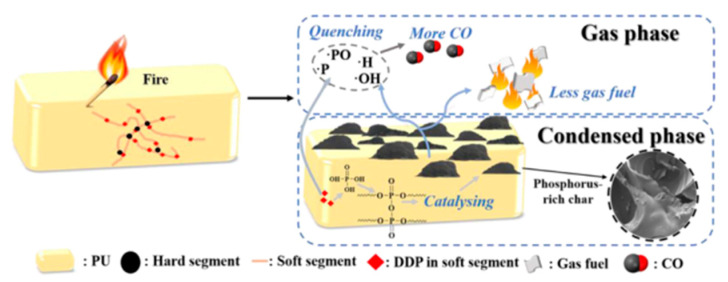
Flame-retardant mechanism of FRPE [64]. Copyright 2014, Elsevier.

Phosphorus-containing FRs exhibited mechanisms, including the gas phase and condensed phase. Rodriguez et al. [64] found that, although THR and PHRR were not significantly reduced, the longer time to ignition (TTI) for flame-retarded polyurethane, based on PPO with only 0.2 wt. %, phosphorous was observed. The improvement of TTI might be due to the fact that reactive phosphorus compounds led to a decrease in the concentration of reactive radicals and gas fuel in the pyrolysis. Wang and co-workers [67] further analyzed the flame-retardant mechanism of FRPE by TG–IR analysis, disclosing the dual role of FRPE in both gas and condensed phases conclusively (shown in Figure 9). An increase in temperature converted FRPE into phosphine and its derivatives, giving off PC• and PO• radicals to delay or interrupt the combustion. Meanwhile, the phosphoric acid and poly-phosphoric acid, which was also produced by FRPE, promoted the dehydration and carbonization of the matrix and increased the residual char rate and ignition time.

To fulfill the environmental protection requirement, Liu [69] has successfully fabricated a degradable waterborne polyurethane that not only possesses both flame retardancy and high tensile strength (up to 35 MPa) but also can be degraded after being buried in the soil. These polyurethanes were made from degradable soft segments—such as polycaprolactone diol, Exolit^®^ OP 550, and castor oil—that originated from bio-based sources. This breakthrough presented a sustainable solution to reduce the reliance on petroleum-based resources.

Further research should focus on optimizing the molecular design and formulation of phosphorus-based flame retardants for PUE, aiming to enhance their flame retardancy while addressing environmental concerns and maintaining desirable physical properties.

## 5. Phosphorus-Heteroatom Synergistic Flame Retardants

Synergistic flame retardants refer to adding two (or more) flame retardants or introducing two (or more) flame-retarded elements to the polymer. Compared with P-FRs, phosphorus–heteroatom flame retardants (heteroatoms including nitrogen, silicon, boron, sulfur, etc.) exhibit mutual synergistic effects, and they have been extensively applied due to their eco-friendliness and high efficiency [21].

### 5.1. P-N Synergistic Flame Retardants

Phosphorus-nitrogen flame retardants (P-N FRs) are usually regarded as one of the most desirable candidates due to their superior advantages, including less pollution and higher flame-retardant efficiency [20,32]. Among the most typical P-N FRs, APP (ammonium polyphosphate) was already used in TPU for years, and it is still evolving with various P-to-N ratios [70]. With the addition of P-N FRs into the polyurethane, the T_5%_ decreased in most cases, and the char residual increased [71]. The P-N synergistic flame-retardant mechanism is similar to P-FRs, including both the condensed phase and gas phase. Usually, P-N synergistic flame retardants were incorporated into TPU as chain extenders or polyols, while a P-N-containing molecule with single OH group was also able to be grafted into thermoset polyurethane elastomers in a few reports [72]. Next, we are reviewing mainstream cases with P-N synergistic flame retardants extending TPU chains (as chain extenders or polyols).

#### 5.1.1. P-N Synergistic Chain Extenders

**Figure 10:**
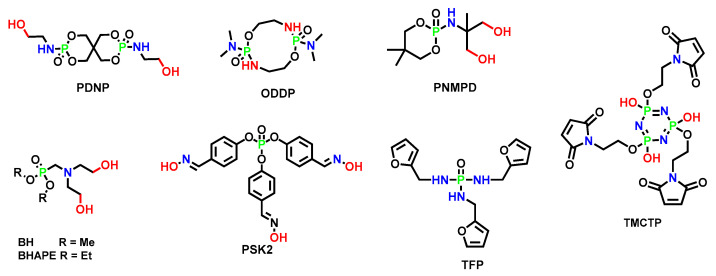
The structure of P-N synergistic chain extenders, PDNP, ODDP, PNMPD, BH, BHAPE, PSK-2, and TMCTP [73,74,75,76,77,78,79].

Table 10 summarizes a variety of P-N synergistic chain extenders, including phosphonate molecules containing tertiary amine groups (BH), phosphor-amide molecules with P-N bonds (ODDP), and phosphazene molecules with P = N (TMCTP), whose structures are shown in Figure 10.

**Figure 11:**
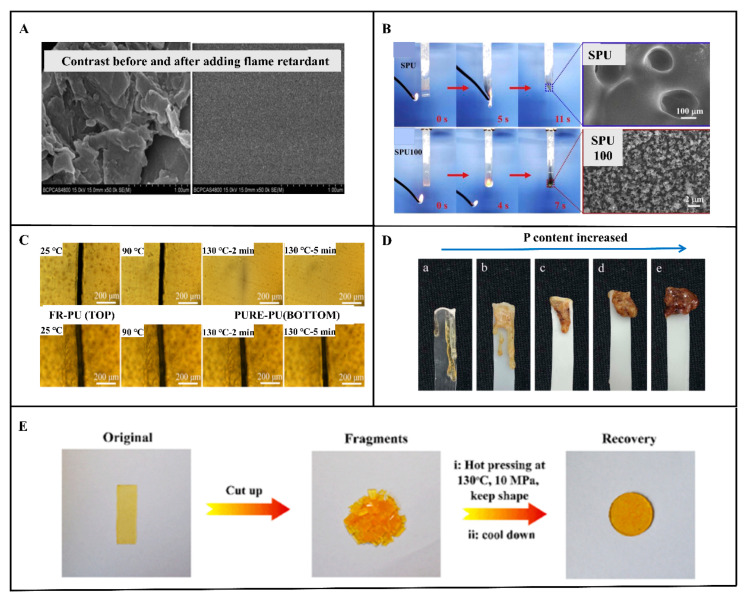
(**A**) SEM image of char residue for polyurethane based on ODDP [80]; Copyright 2015, John Wiley and Sons. (**B**) The SEM photos of polyurethanes based on normal chain extender and BH after vertical burning test [81]; Copyright 2021, Elsevier. (**C**) Self-healing behaviors of polyurethane based on TMCTP [79]; Copyright 2021, American Chemical Society. (**D**) Anti-dripping effect of BSPB on FR-PU, whose molecular structure is shown in Figure 13 (with BSPB content increasing from a–e) [82]; Copyright 2017, Society for Plastic Engineers. (**E**) Recyclability demonstration of MPUF based on TFP [78]. Copyright 2020, Elsevier.

The phosphorous component in P-N synergistic FRs is usually reported to be responsible for the condensed phase flame-retarding behavior. Luo et al. [74,80] synthesized macro-cyclic ODDP and the corresponding polyurethane (DPWPU). The microstructure of the char for DPWPU after burning (Cone Calorimetry test) was displayed (Figure 11A) to be continual and rugged, and this compact char layer could effectively inhibit oxygen transmission and heat diffusion during combustion. In addition, the phosphorus content in the residues grew with the increasing amount of ODDP employed in polyurethane, which indicated that ODDP played an active role in the condensed phase during the decomposition. Wang et al. [81] analyzed the vertical burning test of polyurethane, based on BH, before and after flame-retardant treatment (Figure 11B), finding that the droplet phenomenon was significantly suppressed. Meanwhile, when introducing BH into the polyurethane (SPU), the surface of char residue displayed a rough and porous protective char layer consisting of dense submicron particles. This phosphorus-rich char layer finally contributed to the condensed phase flame-retardant mechanism of BH.

Nitrogen-containing moiety in P-N synergistic FRs endows the TPU with various functions since amine or N-heterocycles are common synthetic handles. Long-term mechanical properties are especially significant functions for PUE, resulting in extensive study on their self-healing behaviors. Self-healing polymers, coined as novel high-performance materials with extended life spans, are divided into intrinsic and external-aid types [83]. The number of healing cycles for external-aid self-healing materials is limited. Thus, intrinsic self-healing materials, relying on dynamic bonds, become a hot spot in recent years [84,85,86]. Various covalent bonds (reversible Diels–Alder reactions, acylhydrazone bonds, and disulfide bonds) and non-covalent bonds (hydrogen bonds, metal coordination bonds, and ionic interactions) have been employed to construct self-healing polymers [87].

The stability and service life of flame-retarded polyurethanes are also significantly improved by introducing dynamic bonds to turn them into self-healing materials and, ultimately, achieve the goal of sustainable development and a reduction in safety hazards simultaneously [88]. Pan et al. [77] successfully synthesized a phosphorus/nitrogen-containing oxime chain extender (PSK_2_) and introduced it into PUE to obtain a transparent, flame-retarded, self-healable (at elevated temperature), and mechanically strong PSK_2_-PUE. The polymer exhibited high tensile strength and good flame retardancy with an LOI of 29.2% and a UL-94 V1 rating. The dynamic exchange of hydrogen bonds and the trans-esterification/trans-carbamoylation of oxime-carbamate endowed PSK_2_-PUE with malleability and self-healing ability. After the PUE sample was cut-off, comparatively limited self-healing at room temperature was demonstrated after 24 h, and the mechanical properties recovered to 60% of the original value after 72 h. Unfortunately, further prolonging healing time or increasing temperature had no positive effects on enhancing the healing efficiency.

Tri-maleimide end-capped cyclotriphosphazene flame retardant (TMCTP) and the corresponding polyurethane (FR-PU) were synthesized by Wang et al. [76,79]. Figure 11C clearly showed that the razorblade crack on the surface of FR-PU gradually healed at 130 °C, and the crack totally disappeared after heating at 130 °C for 5 min. This phenomenon occurred because mobile pre-polymer chains were produced through retro-DA reactions and entangled under high temperatures. Moreover, the introduction of TMCTP significantly promoted the flame-retardant property of FR-PU, benefiting from the phosphorus-containing moieties (phosphates and phosphonates) generated during combustion. The same research group [78] also introduced the phosphor-amide-based flame-retardant TFP (structure showed in Figure 11E) as a cross-linking agent into the polyurethane elastomer (MPUE) to construct a dynamic network, so that MPUE was both self-healing (through reversible Diels–Alder reaction) and flame retardant. The tensile strength of MPUE was significantly improved (14.84 Mpa to 37.11 Mpa), and the self-healing efficiency was higher than 90%. Compared with the unmodified thermoset polyurethane elastomer, MPUE also showed better thermal stability and a limiting oxygen index of 28.5%.

**Figure 12:**
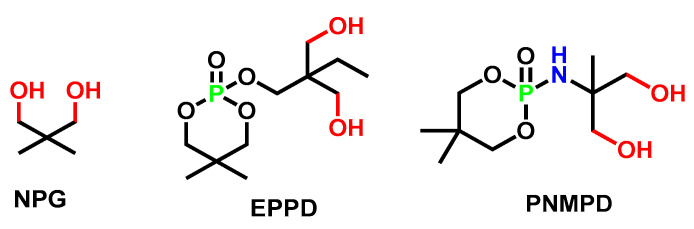
Chemical structure of NPG, P-FRs EPPD, and P-N FRs PNMPD [60,75].

Fan et al. [60,75] systematically compared P-FRs (EPPD) and P-N synergistic FRs (PNMPD). EPPD and PNMPD had similar structures (shown in Figure 12); however, the thermal stability and flame retardancy of the corresponding PUEs were different, as shown in Table 11. The tensile strength of EPPD-based polyurethane was higher (which could be caused by lower contents of flame-retarding units), while PNMPD-based polyurethane maintained good hydrolysis resistance. Meanwhile, the flame-retardant performance of PNMPD-based polyurethane was more excellent since P-N synergistically formed a rugged carbonaceous layer as a physical insulation barrier for underlying polyurethane to impede heat, oxygen, and flammable gas.

**Figure 13:**
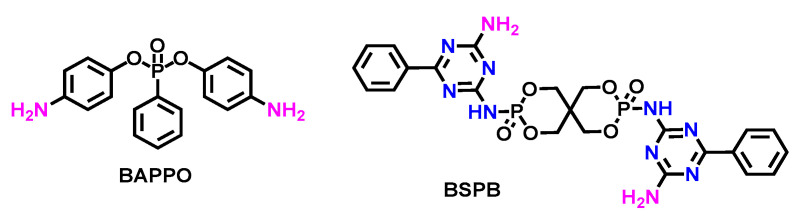
Chemical structures of diamino chain extenders, BAPPO and BSPB [82].

P-N synergistic di-amino chain extenders had the similar flame-retarded behaviors as those di-alcohol analogues in Figure 13. A reactive intumescent phosphorus-nitrogen flame retardant (BSPB) was successfully synthesized by Fan et al., and it was covalently conjugated onto the backbone of waterborne polyurethane (WPU) [82]. The results indicated that, with the increase in BSPB content from 0 to 8 wt. %, the LOI value of polyurethane increased from 18.6 to 27.3%, and the thermal stability decreased while the char residue ratio was promoted (as shown in Table 12). In Figure 11D, it was shown that the outer compact intumescent char formed protective shields to inhibit the spread of heat and flame effectively, thus preventing the dripping of burning molten mass, an inherent problem that is unsolved in most approaches to flame-retardant polyurethanes.

P-N synergistic FRs also affected mechanical properties of TPU. L. Aras et al. [89] synthesized the diamino-phosphate chain extender, BAPPO, and applied it to polyurethane (BAPPO-PU). The hardness and the wear resistance of BAPPO-PU were improved, and the LOI increased to 27%. Besides, BAPPO-PU also passed the mandrel bending and impact resistance tests without any cracks forming on the coating surface. However, incorporating BAPPO into the structure deteriorated the gloss properties, which should be attributed to the change in the chain conformation caused by the mixture of different chain extenders.

#### 5.1.2. P-N synergistic Reactive Polyols

**Figure 14:**
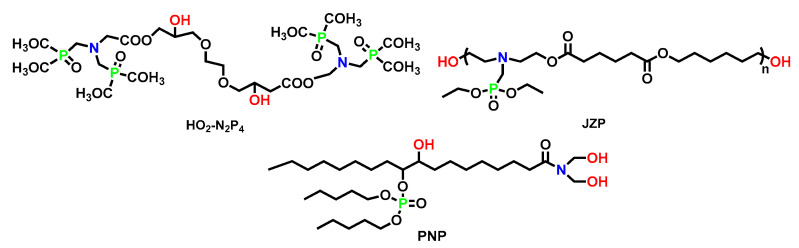
Chemical structures of HO_2_-N_2_P_4_, JZP, and PNP.

**Figure 15:**
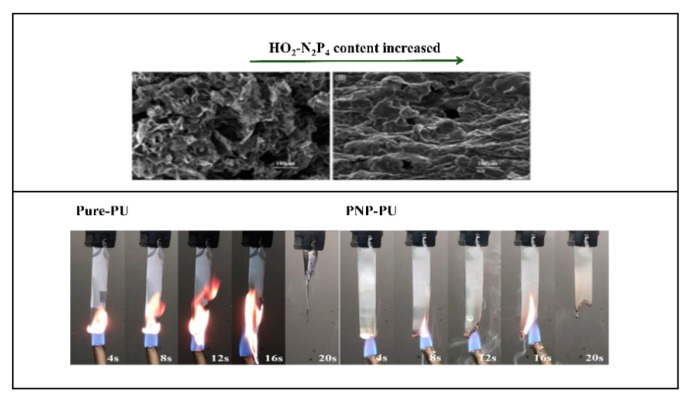
Carbon layer morphology before and after incorporating HO_2_-N_2_P_4_ (**Top**) [90]. Copyright 2022, John Wiley and Sons. Vertical combustion test of PU before and after incorporating PNP (**Bottom**) [92]. Copyright 2021, John Wiley and Sons.

P-N synergistic polyols are typically composed of both tertiary amine and phosphate functional groups within the repeating unit (whose structures are shown in Figure 14 and the flame retardancy effects are displayed in Table 13). For example, a reactive phosphorus and nitrogen-containing monomer (HO_2_-N_2_P_4_) was synthesized, and then incorporated, into polyurethane at various ratios via copolymerization by Cui et al. to slow down the thermal decomposition and reduce the flammability [90]. As shown in Figure 15 (TOP), the char layer of original PU was rough, with cracks and holes, resulting in heat transfer to the interior. After introducing HO_2_-N_2_P_4_, the carbon layer was uniform and complete, and the surface was smooth and without cracks.

Jiang et al. [92] made significant contributions to solving the droplet problem in the polyurethane field by introducing phosphorus-containing polyols (PNP). From the comparison in Figure 15 (Bottom), it could be seen that the droplet behavior was reduced, which was difficult to achieve, although very crucial for reducing the hazard. When the content of PNP was 37.5 wt. %, the tensile strength of composite PU was about two times its original value (tensile strength increased from 2 Mpa to 4.2 Mpa). This phenomenon was caused, mainly, by the fact that tri-functional P/N polyols increased the cross-linking density. Overall, PNP brought great flame-retardant property and mechanical property with high-hydroxyl value and moderate viscosity, making it a promising novel soft segment for high performance PU elastomers.

#### 5.1.3. P-N Synergistic DOPO-Based Flame-Retarded Polyurethane

**Figure 16:**
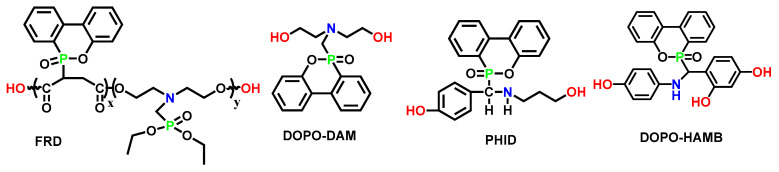
Chemical structures of DOPO-based flame retardants, FRD, DOPO-DAM, PHID, and DOPO-HAMB [76,94,95,96].

DOPO is a widely used and efficient phosphorus-based FR. The active P-H bonds facilely dissociate, and the nucleophilic P is able to add to C=C and C=N bonds to form new C-P bonds [12,93]. A wide variety of DOPO-derived FRs have been prepared. With the phosphaphenanthrene structure, DOPO and its derivatives displayed high thermal stability, excellent flame retardancy in both the condensed phase and gas phase, and they were commonly used as additive flame retardants in various resins, as well as in polyurethanes. Table 14 summarizes the flame retardancy of four DOPO-based P-N synergistic FRs, FRD, DOPA-DAM, PHID, and DOPO-HAMB, and their chemical structures are shown in Figure 16.

P-N synergistic FRs, based on DOPO, could effectively enhance both flame retardancy and mechanical performance. Wang et al. [76] developed a novel polyester diol FRD, which was incorporated into polyurethane backbone, to prepare a series of FRWPU. The resulted polymer materials reached V-0 rating with a LOI value of 30.5% and a remarkable decrease in HRR and THR compared to the pure polyurethane. Meanwhile, the tensile strength of FRWPU-5 (5wt.% FRD) increased significantly from 19.7 to 30.6 Mpa, while the elongation at break increased from 611.1 to 756.4%, indicating that the copolymer structures of flexible phosphate and rigid DOPO moiety coordinated well to contribute higher strength and flexibility in the tensile process. However, the tensile strength of FRWPU-7 (7wt.% FRD) decreased slightly, possibly owing to the unwell-distributed rigid DOPO moieties in polyurethane.

**Figure 17:**
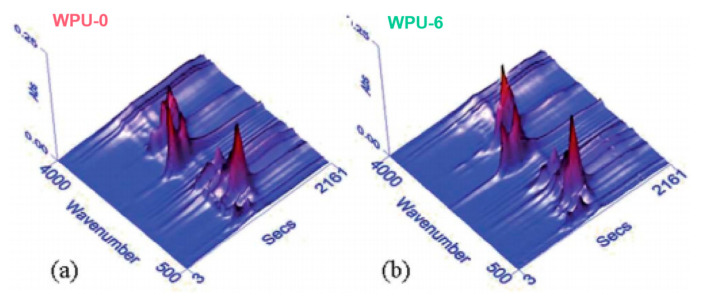
Three-dimensional TGA-FTIR spectra of volatilized products for WPU-0 and WPU-6 (**a** and **b** represent PU (WPU-0) and flame-retarded PU (WPU-6), respectively) [94]. Copyright 2019, Royal Society of Chemistry.

Wang et al. [94] synthesized DOPO-DAM and used it as a chain extender in polyurethane (WPU). From Figure 17, it could be inferred that the composition of decomposition gas products for WPU-6 was similar to that of WPU-0. The new absorption peaks around 1268 cm^−1^ and 1002 cm^−1^ corresponded to P=O and P-O-C, respectively. Meanwhile, the nonflammable gas NH_3_, with a characteristic peak around 928 cm^−1^, was detected. Thus, the gas-phase flame-retardant mechanism was proposed for DOPO-DAM. Compared with WPU-0, a more rigid and thicker porous carbon layer was generated on the surface of WPU-6, forming a protective shield. As a result, the PHRR and THR were significantly reduced. Together with the clues of release of inert gases after the decomposition, it reflected an intumescent flame-retardant mechanism. It was also clearly demonstrated that, in a P-N synergistic flame retardancy system, the nitrogen component usually generated non-flammable gas, while phosphorus exhibited a more vital role in condensed phase.

With two conjugated benzene rings as the core structural motif, DOPO owns the capability of absorbing UV light effectively. Inspired by this feature, Zhang [96] synthesized a novel flame retardant, DOPO-HAMB, via addition of the P-H in DOPO onto aromatic imines. The resulting polyurethanes, FRWPU, exhibited improved flame retardancy and UV-shielding properties while preserving their inherent mechanical strength. In addition, DOPO-HAMB could suppress melt dripping and smoke release during combustion, and these enhancements were attributed to the cooperation between DOPO and aromatic Schiff-based derivatives with both gas-phase and condensed-phase flame-retardant mechanisms. These insights will help advance the field of coatings, adhesives, and treatment agents since PUEs are constantly used as the base materials in them and subjected to long-term UV aging.

### 5.2. Other P-Heteroatom Synergistic Flame Retardants

**Figure 18:**
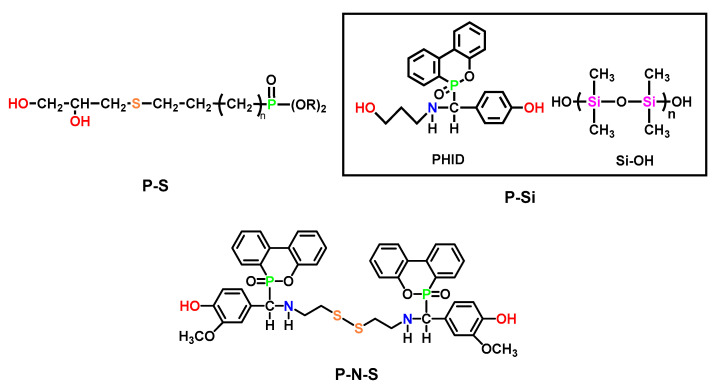
Structures for P-S, P-Si, and P-N-S cooperative flame retardants.

In addition to P-N synergistic flame retardants, phosphorous can also play a synergistic role in polyurethane together with other heteroatoms, such as sulfur, silicon, etc. [97] (Typical examples are shown in Figure 18).

There were limited numbers of examples on P-S synergistic FRs with moderate flame-retardant efficiency. Khatib [98] facilely synthesized a diol chain extender containing P and S by thiol-ene “click reaction” and applied it to flame-retarded polyurethane. With the increase in chain extender content, the char residue and limiting oxygen index (LOI) increased compared to normal polyurethane.

TPU based on P-N-Si synergistic FRs exhibited excellent performance in both flame retardancy and tensile strength. Fan et al. [95] compared the flame retardancy of Si-FR, P-N synergistic FR, and P-N-Si synergistic FR, and the details were shown in Table 15. The results demonstrated that the synergistic effect of PHID with hydroxyl-terminated polysiloxane (HO-Si) could generate a more compact char to protect the underlying polyurethane, while the decomposition of PHID produced PO• radicals to interrupt the combustion reaction. Besides, HO-Si produced a special insulating SiO_2_ layer to improve the thermal oxidative stability. The synergistic effects of these factors contributed to the designable flammability of the WPU coating. Further investigation revealed that Si-containing units could also efficiently balance out the usual loss of other properties in flame-retarded WPU [99,100,101]. For example, both the tensile strength and elongation of FR/Si-WPU, at break, were found to increase, which was hard to achieve for flame-retarded polyurethane. Similar changes in mechanical properties were observed in another case of P-N-Si synergistic-flame retarded TPU, as reported by Song and Xu et al. recently [102].

**Figure 19:**
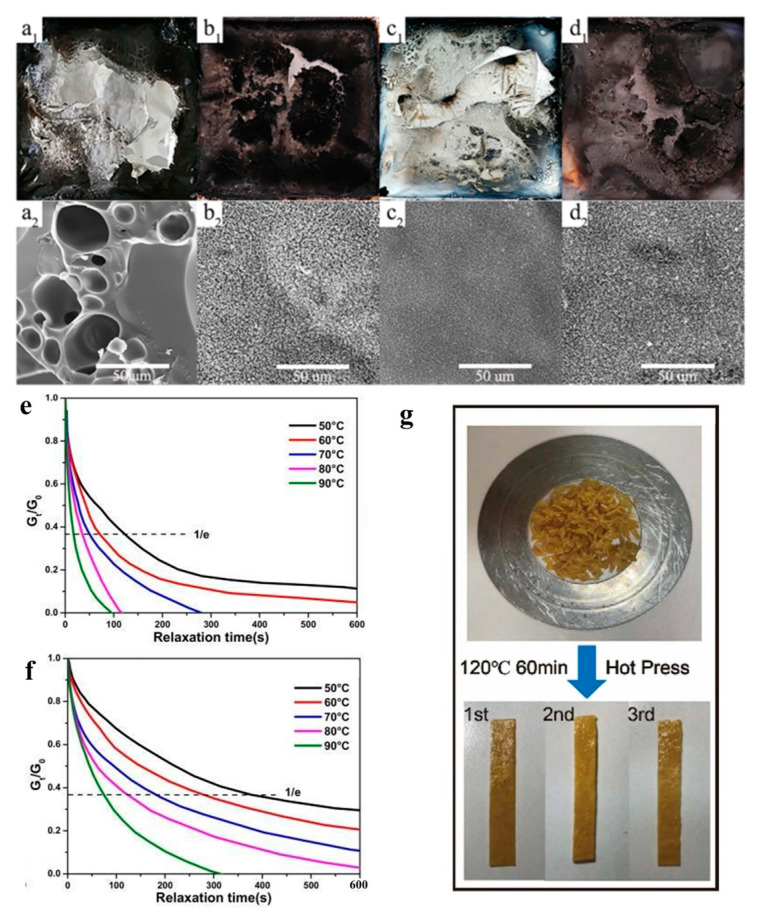
Photographs of char residue from (**a1**) WPU, (**b1**) FR-WPU, (**c1**) SI-WPU, (**d1**) FR/SI-WPU, and respective SEM pictures (**a2**–**d2**) [95]. Copyright 2021, Elsevier. Stress–relaxation curves of VDPU-0 and VDPU-20 (**e**–**f**); optical images of the reprocessing performance of the VDPU-20 by hot pressing (**g**). Copyright 2023, Elsevier.

The diversification of chain extenders for polyurethane elastomers (PUEs) has allowed for the incorporation of multiple functionalities, expanding their range of applications. Zeng [103] prepared P-N-S flame-retarded PU with 30.2% LOI value and passed the V-0 vertical burning test. A novel vanillin-based chain extender and its DOPO adduct were designed, resulting in a polyurethane (PU) with dual-dynamic covalent bonds (disulfide and imine), enabling self-healing and recyclability capabilities (Figure 19g). It is important to note that, as the content of rigid DOPO increased, the dynamic properties of the polyurethane network were compromised, as proven by a longer stress–relaxation time at the same temperature (as depicted in Figure 19e,f). However, despite this compromise, the dynamic network could still be effectively recycled through hot pressing within a time frame of 60 min at a temperature of 120 °C.

Above all, exploring synergistic combinations of phosphorus-based flame retardants with other elements or technologies can lead to enhanced flame-retardant performance and multifunctionality in PUE materials.

## 6. Perspective on Future Development of P-FRs-Based TPU

### 6.1. Design of P-FRs Inspired from the Intrinsic Flame Retarded Polyurethane Foam

After extensive investigation and analysis of a large number of literatures, we found that phosphorus-containing diol (used as chain extenders) and phosphorus-containing polyols were widely used in polyurethane foams [104]. Table 16 sums up five representative P-containing flame retardants used in polyurethane foams, which could also be considered as the potential intrinsic flame retardants for polyurethane elastomers [105]. For example, P-N FRs PNMPD mentioned in Section 5.1 had similar structure and P content to HAMPP. However, the flame-retardant performance of PNMPD on PUE was more phenomenal at a similar concentration, implying the great potential of other reactive flame retardants already applied in foams.

Non-halogenated FRs HAMPP was synthesized and applied in polyurethane foam by Li et al. [106]. With a cyclic phosphate structure, HAMPP could readily carbonize the polymer materials to inhibit or prevent further combustion upon flame through both the gas and condensed phase mechanisms. Besides, the additions of these FRs maintained the mechanical properties of the foam. Bhoyate [107] synthesized phenyl phosphonic acid and propylene oxide-based reactive FR polyol (PPA-PO-polyol), and they used them along with limonene-based polyol for the preparation of FR polyurethane foam. The addition of PPA-PO polyol effectively increased the closed cell content (more than 96%). Moreover, 1.5 wt.% of phosphorus (P) content in polyurethane reduced the self-extinguishing time of the foam from 81 s (28% weight loss) to 11.2 s (9.8% weight loss). Ramos [110] reported the synthesis of aminophosphonated polyols and the corresponding polyurethane. As can be seen in Table 16, with phosphorus contents increasing from 0 to 3%, all the PHRR, THR, and TSR are significantly reduced, especially with the decrease in 122% for TSR, possibly due to the presence of metal ions, which is essential for hazard reduction in flame-retarded polyurethane foam (FPUF). FPUF possessed a huge market share in building materials, so the reduction in smoke production could provide the safety guarantee when people escaped from the fire. Similarly, reducing smoke would also stand out as one of the key preventions for fire hazards of polyurethane elastomer that was applied in construction and automobile environments.

**Figure 20:**
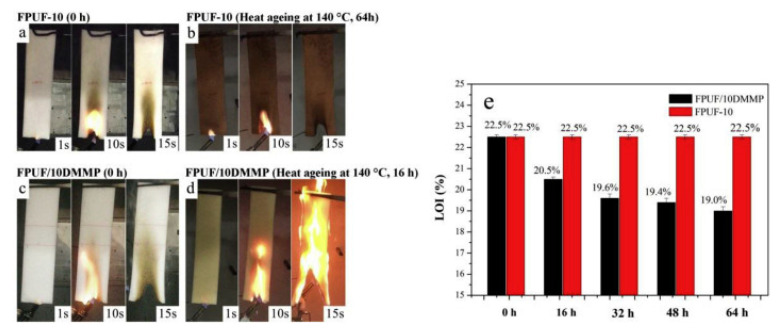
(**a**–**d**) The vertical combustion test and (**e**) LOI of additive flame-retarded FPUF/10DMMP [103] and reactive flame-retarded FPUF-10 before and after the aging test [108,109]. Copyright 2018, Elsevier.

Reactive flame-retarded foam networks were also proven to avoid the possible migration and leaching of FRs, leading to better compatibility between FRs and the foam matrix. Wang et al. [108,109] compared the aging resistance of additive flame retardant (DMMP) and reactive flame retardants (PDEO and DMOP as chain extenders) in polyurethane foams. As presented in Figure 20, additive-type flame-retarded polyurethane foam (FPUF/10DMMP) showed self-extinguishing behavior before the thermal aging test. However, after being subjected to 140 °C for 16 h, the FPUF/10DMMP failed to pass the vertical burning test, and the LOI value decreased from 22.5% to 20.5%. The reason for the decrease was attributed to the migration of flame retardants during aging. In contrast, reactive-type flame-retarded polyurethane foam (FPUF-10) still maintained its flame retardancy after the thermal aging test at 140 °C for even 64 h (Figure 20). This indicated that the reactive flame retardant was more persistent and durable than the additive flame retardant, which would also be applicable for polyurethane elastomer and echoed the essence of development for reactive phosphorous flame-retarded TPU.

Cautions should be taken when borrowing the principles applied in PU foams to design PUE. First, polyurethane foam is a cross-linked polymer material. The chain extender and polyol used to construct them usually have more than three functionalities, so the number of hydroxyl groups, as well as the solubility and compatibility of the flame retardants, should be considered when being applied in polyurethane elastomers. Furthermore, the limiting oxygen index (LOI) of flame-retarded polyurethane foam is generally less than 24%, and the same P-FRs employed in polyurethane elastomers have higher LOI values (higher than 25%), potentially caused by higher open cell contents, resulting in more exposure to air compared with elastomers.

### 6.2. Design of P-FRs Inspired from the Intrinsic Flame Retarded Epoxy Resin

**Figure 21:**
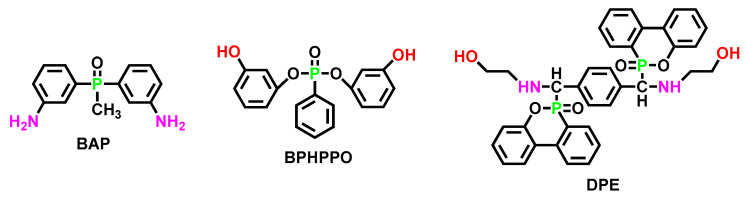
Chemical structures of phosphorus-containing di-alcohol/phenol/amine, including BAP, BPHPPO, and DPE.

Phosphorus-containing di-alcohol (di-amine) used to cure epoxy resins (via nucleophilic ring opening) can also function as a reactive chain extender to synthesize polyurethane elastomers. Figure 21 summarized three phosphorus-containing di-nucleophilic flame retardants applied in epoxy resins.

With higher content of BPHPPO or DPE [111,112] in epoxy resin, the T_5%_ decreased, and the char residue increased. This trend was similar to that of phosphorus-containing polyurethane elastomers. Indeed, the LOI value of the flame-retarded epoxy resin reached 34% excellently.

**Figure 22:**
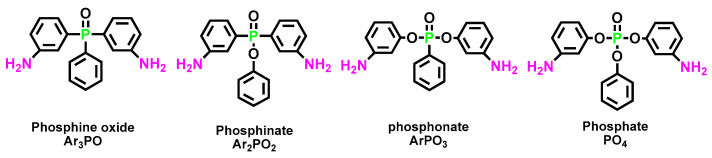
Structures of phosphonate FRs with different P oxidation states.

Braun et al. [59] compared the thermal degradation behavior and flame retardancy of phosphorus-containing carbon-fiber-reinforced epoxy resins with different oxidation states of phosphorous (shown in Figure 22). With a higher oxidation state, the char residue increased from 24% to 30%, while the LOI and UL-94 class decreased. These clearly indicated that the flammability performance was mainly dictated by gas phase action, whereas the difference in the charring of the materials played a negligible role, which was attributed to the already-high char yield of the carbon fiber component. Thus, the design of a flame retardant for reinforced polyurethane elastomer composites should also focus on the gas phase mechanism.

There are also more advanced strategies of combining phosphorous moieties with other elements to realize the goal of flame retardancy in epoxy resin. By integrating metal organo phosphonates, outstanding flame retardancy and smoke suppression performances were achieved by Chen and Wang et al. [113], along with improved performances in UL-94 and LOI tests. This novel strategy of combining valid components through coordination bonds (based on diphenylphosphinodithioate ligands) towards flame retardancy and smoke inhibition also provided unique insights into designing intrinsic fire-safety polyurethanes with comprehensive properties.

Among the essential challenges for the employment of the P-FRs already applied in epoxy resins is whether they could be dissolved in the process of bulk polyurethane preparation, which usually occurs at a lower curing temperature (lower than 100 °C). If this problem was solved, the design of intrinsic phosphorous flame-retarded TPU would make steps into a new era.

### 6.3. More Environmentally Friendly Flame Retarded Polyurethane with Biological Based Material

**Figure 23:**
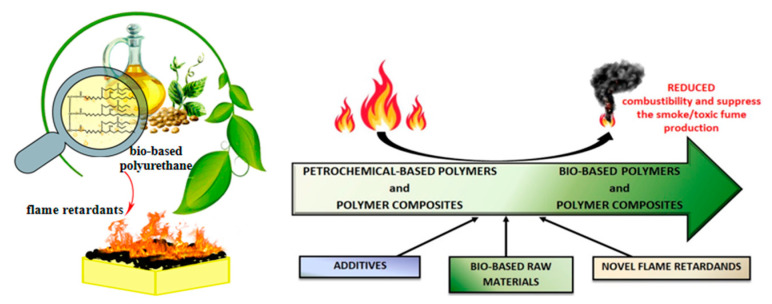
Concept of flame retardancy of bio-based polyurethanes [117]. Copyright 2014, Elsevier.

Bio-based flame retardants have a wide market due to their non-toxicity, environmental protection, low price, and other characteristics (shown in Figure 23) [114]. Therefore, the development of bio-based polyurethanes with flame-retardant properties has been at the center of attention in recent years [31,115,116]. Bio-based polyurethanes are usually prepared from renewable sources, such as vegetable oils, cardanol, lignin derivatives, isosorbide, phosphorous-containing phytic acid, and so on.

Soybean oil is one of the most widely explored bio-based raw materials for the synthesis of PUs [117]. Aynur et al. [118] synthesized phosphorus-containing polyols from epoxy soybean oil and applied them in polyurethane foam, with the LOI increasing to 26.4%. Lignin is presently the second largest biopolymer source in nature after cellulose [119], which can replace petroleum-based polyols to provide a new choice for renewable polyurethane. Wang [120] synthesized a novel lignin-based phosphorus-containing flame retardant (LMD), and the LOI of the corresponding polyurethane reached 29.9%, showing excellent flame retardancy. However, the bio-based chain extender only weighed up 20% of the total materials. It was noteworthy that the bio-based Pus, developed by Aynur et al., had bio-based constituents taking more than 70% of the total weight, in contrast to many reports stating the development of bio-based polyurethanes, while only bio-based chain extenders were used. With the continuous efforts of developing phosphorus-based flame retardant, including both modifying general bio-based components (lignin grafted with phosphate) and taking advantage of phosphorous-inherent compounds (phytic acid), the future for bio-based flame-retarded polyurethane is bright [121].

### 6.4. Functional Flame-Retarded Polyurethane

#### 6.4.1. Application of Flame-Retarded Polyurethane in Polyelectrolyte

Lithium ion batteries (LIBs) with high energy density, low maintenance, and self-discharge are widely used in electric vehicles (EVs) [122]. However, frequent fires and explosion-associated accidents limit their further widespread applications. Currently, most commercial lithium-ion batteries use liquid electrolytes, including components with low flash points (<40 °C), and their vapors readily catches fire, bringing severe safety risks. Solid electrolytes, including solid polymer and solid inorganic electrolytes, are regarded as ideal candidates for a lithium ion battery, due to their good safety performance and high temperature resistance [100].

**Figure 24:**
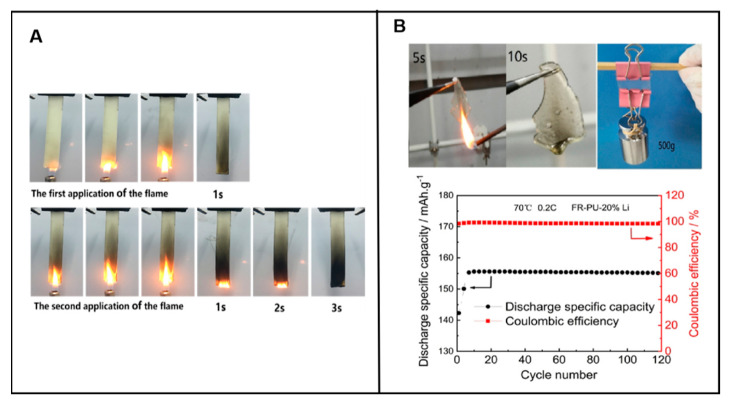
(**A**) UL-94 test performance of FR-SPE grafted with DOPO [123]. Copyright 2022, American Chemical Society. (**B**) Flame retardancy, mechanical strength, and discharge-specific capacity of FR-PU [120]. Copyright 2022, Elsevier.

Flame-retarded polyurethane elastomers have been demonstrated as one type of solid polymer electrolyte (SPE), while the electrochemical properties of the assembled battery are not reduced. Lv et al. [123] successfully designed a polyurethane-based flame-retarded solid polymer electrolyte (FR-SPE). The grafting of DOPO endowed the FR-SPE membranes with a high fire-retardant efficiency, reaching V-0 level in the UL-94 test (Figure 24A). In addition, no adverse effect on the cycle performance of batteries with graphite electrodes was observed. Chen et al. [124,125] enhanced the safety of lithium batteries by developing another phosphorus-containing flame-retarded polyurethane (FR-PU) solid electrolyte. As shown in Figure 24B, the FR-PU not only displayed excellent flame retardancy with the limit oxygen index value of 27.6% but it also exhibited sufficient mechanical strength, heat resistance, and superior discharge specific capacity after certain cycle numbers. Meanwhile, Song et al. [126] successfully obtained stretchable, ultra-tough, and intrinsically self-extinguishing polyurethane elastomers as substrate for strain sensors. This showed that the application of flame-retarded polyurethane was increasingly matching the high-tech industry, especially in the form of surface coating.

Consequently, when the lithium-ion battery encounters high temperatures in a fire disaster, flame-retarded PUE coating rapidly carbonizes and releases non-flammable gas to block the external oxygen and cut off the heat transfer, thus effectively interrupting the fire. There are sufficient reasons to believe the application of intrinsic flame-retarded polyurethane in lithium-ion batteries has a broad development prospect.

#### 6.4.2. Self-Healing Flame-Retarded Polyurethane

Common polyurethane is inevitably damaged, generating small cracks during its molding, processing, and use [112,127]. These cracks will accelerate the stress diffusion until the material fractures, which ultimately reduces the service life of the material. Therefore, in order to maximize the utilization of resources, the development of recyclable and self-healing polyurethane is essential [128]. Thermoplastic polyurethane is intrinsically self-healing since the interchain interactions could be broken at an elevated temperature, while thermoset polyurethane elastomer, with higher mechanical stability, is still considered to be non-self-healing after the curing process is over. Flame-retarding dynamic bonds have been gradually developed and applied in multiple types of thermoset materials, such as epoxy resin and vinyl resin [129,130,131,132,133,134,135]. In the future, it is foreseeable to introduce dynamic groups (both covalent and noncovalent) into the privileged polyurethane thermoset and endow it with flame-retardant properties through incorporating flame-retarding elements (phosphorous as the most typical example) or novel flame-retarding mechanisms, as Pan et al. have done [77].

With the rapid development of information technology and artificial intelligence, especially the advent of the 5G era, people have more advanced requirements for electronic products, such as flexibility and wearability. Traditional metal or semiconducting electronic sensors are usually hard to bend. Its limited elastic strain range cannot meet the demand of smart applications in the future. Thus, wearable device takes more advantage of the tougher polymer-based materials, especially polyurethanes with superior mechanical properties, compared with polydimethylsiloxane (PDMS) [136], as well as high chain mobilities (composed of soft segments with low T_g_) for ion/electron conducting. Li et al. [137] composed a stretchable strain-sensor at large strain from TPU, MXene, and chitosan (CS), and they explored its application in electronic skin and motional detecting. Similarly, Liu et al. [138,139] prepared self-healing flexible pressure and stress–strain sensor based on the composite of TPU with silver nanowire, graphene, carbon nanotube, and other nano-materials. The majority of current studies centered on the self-healing behavior and conductivity of the electronic devices based on polyurethane without touching the flame-retardant property. However, many of these wearable devices are in direct contact with human skin, and severe life hazards will be caused as soon as they catch fire.

Thus, endowing polyurethane with self-healing and flame retarding at the same time will be an essential route to high working efficiency, safety, and long usage.

## 7. Conclusions

Nowadays, P-containing FRs are the most promising substituents for widely used halogenated FRs for polyurethanes, due to their outstanding compatibility with the polymer matrix and their high flame retardancy. A great number of examples, demonstrating various applications of reactive P-containing FRs in PUEs, are summarized in this review. However, the most desirable P-containing flame retardants, in terms of efficiency, sustainability, and compatibility, with the most advanced functions of polyurethane elastomers, still require countless efforts. There are several technical and scientific considerations to be addressed in the future, including environmental friendliness, toxicity control for both the flame retardants themselves and the burning products, flame-retardant persistence, multifaceted fire safety, and recyclability. Besides, melt-dripping, another common phenomenon in the combustion of PUE, demands extra attentions for the design of high-performance reactive P-FRs [140,141]. Meanwhile, PUE can be designed to possess additional functionalities besides flame retardancy. By combining P-FRs with other additives or modifiers, PUE can exhibit properties such as self-healing, self-extinguishing, antimicrobial, or mechanical reinforcement.

Above all, with more efficient synthetic methods developed for preparing P-containing compounds [12,21,41], there is great room for the development of novel reactive FRs suiting for PUE and applied as coating materials in various advanced fields, including sustainable energy, biomedical device, and a new generation of smart materials.

## Figures and Tables

**Table 1:** Classification and comparison of the advantages and disadvantages of various polyols (↑ represents increase and ↓ represents decrease) [8].

Type	Representation	Advantage	Disadvantage
Polyether polyol	PEG	Hydrolytic stability↑, cost↓, flexibility↑	Oxidative stability↓, strength↓, flammability↑, thermal stability↓, oxidative stability↓, cost↑
	PPG		
	PTMEG	Hydrolytic stability↑, strength↑	
Polyester polyol	Aliphatic polyester polyol	Oxidative stability↑, strength↑	Hydrolytic stability↓
	Aromatic polyester polyol	Thermal stability↑, stiffness↑	Flexibility↓
Polyolefin polyol	Polybutadiene Polyol	Low-temperature flexibility↑, solvent resistance↑	Cost↑

**Table 2:** Di-isocyanate classification, advantages, and disadvantages.

Type	Representation	Advantage	Disadvantage
Aromatic di-isocyanate	TDI	High reactivity, low production cost	Photo-oxidative instability
	MDI		
Aliphatic di-isocyanate	HDI	High chemical stability, excellent weather resistance	High production cost, low reactivity
	IPDI		
	HMDI		

**Table 3:** Flame retardancy and mechanical properties of polyurethane based on NPG, EPDD, BDO, and HMCPP.

HS	SS	Chain Extender	Quantity of P Component (wt. %)	T_5%_ (°C)	R_char_ (wt.%)	UL-94	LOI (%)	Tensile Strength (MPa)	Elongation at Break (%)	Ref.
HMDI	PPG	NPG	0	278.1	1.46	No	18.2	16.4	264.1	[60]
		EPDD	0.25	249.4	3.32	V-0	26.6	14.0	402.2	
MDI	PCL	BDO	0	344.9	2.67	V-2	21	28.3	702	[61]
		HMCPP	1.19	325.7	17.7	V-0	31.8	6.07	1235	

**Table 4:** Flame retardancy and thermal stability brought by P-containing chain extenders (alcohols/phenols).

HS	SS	Chain Extender	Content of P Component (wt.%)	T_5%_ (°C)	R_char_ (wt.%)	UL-94	LOI (%)	PHRR (kW/m^2^)	THR (MJ/m^2^)	Ref.
IPDI	PPG	BDO	0	272.0	0.69	-	17.8	727	93.8	[55]
		BPAMPP	0.54	284.3	1.61	-	27.4	610	73.8	
IPDI	PBA	-	0	165.1	2.14	No	19.3	812.3	-	[56]
		THPO	0.44	263.9	4.37	V-0	25	444.7	-	

**Table 5:** Mechanical properties of WPU and flame-retarded polyurethane (PWPU-X) before and after hydrolysis [60].

Sample	Tensile Strength (Mpa)			Elongation at Break (%)		
	Before Hydrolysis	After Hydrolysis	Retention Ratio (%)	Before Hydrolysis	After Hydrolysis	Retention Ratio (%)
WPU	16.4	15.4	93.9	264.1	244.4	92.5
PWPU-3 ^a^	15.6	14.6	93.6	305.5	277	90.7
PWPU-6	15.2	14.1	92.8	339.6	308	90.7
PWPU-9	14.8	13.5	91.2	372.2	334.7	90
PWPU-12	14.0	13.0	92.9	402.2	366.6	91.1

^a^ PWPU-X, X represents the content of phosphorus-containing chain extender in weight percent.

**Table 6:** Performance comparison of polyurethane based on flame-retarded polyols [64,65,66,67].

HS	SS	Quantity of P Component (wt.%)	T_5%_ (°C)	R_char_ (wt.%)	UL-94	LOI (%)	TTI (s)	PHRR (kW/m^2^)	THR (MJ/m^2^)	Ref.
MDI	PPO	0	260	0	-	20	31	-	108	[64]
		1.44	149	2.09	-	30	39	-	83.7	
MDI	OP	0	304.7	3.8	V-2	22.4	24	2232	132.2	[65]
		9.5	212.7	24.37	V-0	25.2	19	643	47.5	
MDI	PCEPEP-PO	0	324.2	7.7	-	26.5	68	570.54	91.77	[66]
		2.58	288.1	34.8	-	28.8	26	549.55	25.5	
MDI	FRPE	0	323	0	V-2	19	85	758	70	[67]
		0.72	288	16	V-0	24	101	664	53	

**Table 7:** Effects of FRPE and the hard segment on the mechanical properties of polyurethane [67].

A	Sample	Tensile Strength (MPa)	Elongation at Break (%)	B	Sample	Tensile Strength (MPa)	Elongation at Break (%)
	PUE	16.3	1016.1		FR-PUE-28 ^b^	25.0	606.2
	FR-PUE-0.14 ^a^	16.2	904.4		FR-PUE-34 ^b^	28.2	642.7
	FR-PUE-0.29 ^a^	17.6	791.6		FR-PUE-37 ^b^	40.4	466.9
	FR-PUE-0.58 ^a^	20.7	693.2		FR-PUE-40 ^b^	38.0	496.1
	FR-PUE-0.72 ^a^	25.0	605.8		FR-PUE-90 ^b^	20.7	703.0

^a^ In FR-PUE-XX, XX represents the content of FRPE. ^b^ In FR-PUE-YY, YY represents the hard segment content.

**Table 8:** TGA comparison of flame-retarded polyurethane and non-flame-retarded REF in nitrogen and oxygen environments [65].

Atmosphere	PU	T_5%_ (°C)	T_50%_ (°C)	T_70%_ (°C)	Char Residue (wt.%)
N_2_	REF	304.7	319.7	424.7	7.28
	FPU-9.5	212.7	297.8	513.3	24.37
O_2_	REF	307.3	397.3	464.8	0.00
	FPU-9.5	217.2	459.2	665.7	30.14

**Table 9:** Effects of different hard segments on the properties of polyurethane EERPPU [68].

	Soft Segment	Hard Segment	M_w_ (Da)	Hardness	T_10%_ (°C)	Char Residue (wt. %)	LOI (%)	UL 94
EERPPU-1	Phospho- polyol	TDI	7907	80	225	10.09	29	V-0
EERPPU-2		IPDI	9016	75	265	8.56	27	V-1
EERPPU-3		HMDI	7612	72	225	8.03	26	V-1
EERPPU-4		MDI	9330	83	279	11.41	30	V-0

**Table 10:** Flame-retardant effects of P-N synergistic chain extenders.

HS	SS	Chain Extender	Quantity of P/N Component (wt.%/wt.%)	T_5%_ (°C)	R_char_ (wt.%)	UL-94	LOI (%)	PHRR (kW/m^2^)	THR (MJ/m^2^)	Ref.
IPDI	PPG	BDO	0/0	274.3	0	No	19.5	1417.6	59.4	[76]
		BHAPE	0.85/0.38	257.6	4.4	V-2	25.5	810	45	
IPDI	PPG	PDNP	0/0	261.2	0	-	19.5	866	61	[73]
			1.61/0.73	244.9	1.62	-	26	705	46	
HMDI	PPG	NPG	0/0	276.9	0.71	No	18.5	1059	71.5	[75]
		PNMPD	1.4/0.63	252.8	5.44	V-0	27.2	586	43.6	
MDI	PTMEG	PSK-2	0/0	271	5.7	V-2	18.7	-	-	[77]
			0.87/3.2	330	8.7	V-1	29.2	-	-	
TDI	PPG	ODDP	0/0	245.6	3.06	V-2	25.4	-	-	[80]
			4.13/3.73	208.2	18.18	V-0	29.8	-	-	
IPDI	PTMEG	BH	0	318.4	0.2	No	-	-	-	[81]
			1.12/0.5	289.9	2.57	V-0	28.3	-	-	

**Table 11:** Comparison of mechanical property and flame retardancy for polyurethane, based on P-FRs (EPPD) and P-N FRs (PNMPD), against polyurethane extended with NPG [60,75].

HS	SS	Chain Extender	Quantity of P/N Component (wt.%/wt.%)	T_5%_ (°C)	R_char_ (wt.%)	UL-94	LOI(%)	Tensile Strength (MPa)	Elongation at Break (%)	PHRR (kW/m^2^)	THR (MJ/m^2^)	Ref.
HMDI	PPG	NPG ^a^	0/0	278.1	1.46	No	18.2	16.4	264.1	-	-	[60]
		EPPD	0.25/0	249.4	3.32	V-0	26.6	14.0	402.2	-	-	
HMDI	PPG	NPG ^a^	0/0	276.9	0.71	No	18.5	15	385.6	1059	71.5	[75]
		PNMPD	1.4/0.63	252.8	5.44	V-0	27.2	8.2	443.4	586	43.6	

^a^ Polyurethane with NPG has two entries since they had different hard segment contents and were taken from two different literatures.

**Table 12:** Effects of P-N synergistic di-amine chain extenders on the mechanical property and flame retardancy [82].

HS	SS	Chain Extender	Quantity of P/N Component (wt. %/wt. %)	T_5%_ (°C)	R_char_ (wt.%)	UL-94	LOI (%)	Tensile Strength (MPa)	Elongation at Break (%)	Ref.
IPDI	PCDL	BDO	0/0	274.3	0.74	No	18.6	15.3	347.2	[82]
		BSPB	0.76/1.71	234.8	4.76	V-0	27.3	26.4	264.3	

**Table 13:** Flame retardancy of P-N cooperative reactive polyols.

HS	SS	Quantity of P/N Component (wt.%/wt.%)	T_5%_ (°C)	R_char_ (wt.%)	UL-94	LOI (%)	HRR (kW/m^2^)	THR (MJ/m^2^)	Ref.
TDI	HO_2_-N_2_P_4_	0/0	266.2	5.71	No	19	491	132	[90]
		6.1/1.4	192.6	35.86	V-0	32	59	35	
TDI	JZP	0/0	-	3.1	No	18	700	-	[91]
		1.75/0.79	-	12.9	V-0	28.6	400	-	
IPDI	PNP	0/0	280	0.02	No	20.4	326	32.2	[92]
		3.68/1.66	312	9.1	V-0	25.5	105	21.7	

**Table 14:** Effects of DOPO-based FRs on the flame retardancy [76,94,95].

HS	SS	Chain Extender	Quantity of P/N Component (wt.%/wt.%)	T_5%_ (°C)	R_char_ (wt.%)	UL-94	LOI (%)	PHRR (kW/m^2^)	THR (MJ/m^2^)	Ref.
IPDI	PPG	BDO	0/0	274.3	0	No	19.5	1417	59.4	[76]
		FRD	0.79/0.25	264.5	5	V-0	30.5	670	39.7	
IPDI	PPG	BDO	0/0	280.1	1.21	-	18.4	976	64.48	[94]
		DOPO-DAM	0.82/0.37	266.6	4.38	-	31	577	42.45	
IPDI	PCDL	BDO	0/0	273	2.28	No	18.2	814	82	[95]
		PHID	0.94/0.43	231	9.18	V-0	25.1	448	62.5	

**Table 15:** A summary of the flame-retardant properties and mechanical properties for P-Heteroatom synergistic flame-retarded polyurethane and reference PU.

Samples ^a^	R_char_ (wt.%)	TTI (S)	THR (MJ/m^2^)	PHRR (kW/m^2^)	TSP (m^2^)	Tensile Strength (Mpa)	Elongation at Break (%)
WPU	2.3	31	82	814	5.3	13.4	429.6
FR-WPU	8.7	38	62.5	448	4.5	16.6	397.2
SI-WPU	3.9	39	66.6	550	4.7	12.8	463.5
FR/SI-WPU	12.6	44	31.6	159	1.9	15.9	435.6

^a^ WPU, FR-WPU, SI-WPU, FR/SI-WPU represent normal polyurethane, P-N flame retarded polyurethane, Si flame retarded polyurethane, and P-N-Si flame retarded polyurethane, respectively.

**Table 16:** Flame retardancy of reactive P-FRs used in polyurethane foams.

Name	Chemical Structure	Quantity of P Component (wt.%)	LOI (%)	UL-94	PHRR (kW/m^2^)	THR (MJ/m^2^)	Ref.
HAMPP	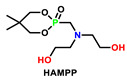	1.2	23.7	V-0	decrease 15.6%	decrease 27.5%	[106]
PPA-PO-polyol	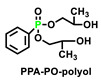	2	-	-	decrease 68.6%	decrease 23.4%	[107]
PDEO	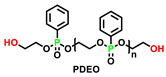	2	23	V-0	No change	62.4%	[108]
DMOP	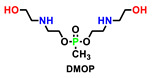	3.7	22.4	V-0	decrease 31.5%	decrease 43.8%	[109]
aminophosphonate polyether polyol	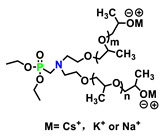	3	-	-	decrease 112.4%	decrease 125%	[110]

## Data Availability

No data were created.

## Chemical Structure and Abbreviation

Chemical structure	Abbreviation	Full name
	Phosphonate diol	Phosphonate diol
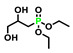
	PAMAD	Phytic acid- 3-(benzyloxy)-2- ((benzyloxy)methyl)-N-(4,6-diamino-1,3,5-triazin-2-yl)- 2-methyl propanamide
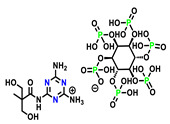
	1,2-P-POSS	1,2-propanediol isobutyl POSS
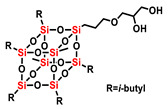
	Diglyceride borate	Diglyceride borate
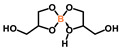
	DiSe	Di(1-hydroxyethylene) Diselenide
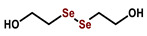
	PEPE	4-(phenylethynyl) di(ethylene glycol) phthalate
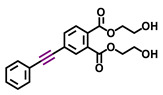
	NPG	Neopentyl glycol
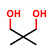
	EPPD	Synthesis of 2-ethyl-2-(2-oxo-5,5-dimethyl-1,3,2- dioxaphosphorinanyl-2-methylene)-1,3-propanediol
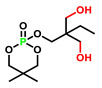
	HMCPP	(3-(2,2-bis(hydroxymethyl)butoxy)-3-oxopropyl)(phenyl)phosphinic acid
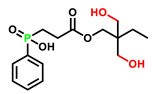
	BPAMPP	bis(4-(2-(4-hydroxyphenyl)propane-2-yl)phenyl) phenyl phosphonate
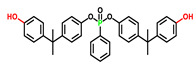
	THPO	tris(hydroxymethyl)phosphine oxide
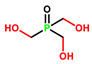
	PPO	(1,3-bis(2-hydroxypropoxy)propane-2-yl)phosphonic acid
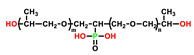
	OP	Phosphorous polyol
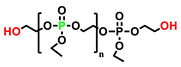
	PCEPEP-PO	2-hydroxyethyl 3-((2-hydroxyethoxy)(phenyl)phosphoryl)propanoate
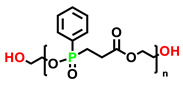

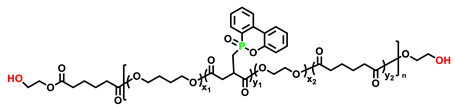

	FRPE	flame-retardant polyester diol
	PDNP	pentaerythritol di-N-hydroxyethyl phosphamide
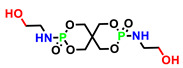
	ODDP	octahydro-2,7- di(N,N-dimethylamino)-1,6,3,8,2,7-di-oxadiazadiphosphecine
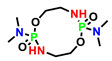
	PNMPD	2-(5,5-dimethyl-2-oxo-2l5 -1,3,2- dioxaphosphinan-2-ylamino)-2-methyl-propane-1,3-diol
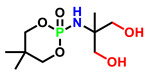
	BH	[bis(2-hydroxyethyl)amino]-methyl- phosphonic acid dimethyl ester
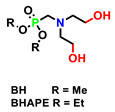
	PSK2	4-((E)-(hydroxyimino)methyl)phenyl bis(4-((Z)-(hydroxyimino)methyl)phenyl) phosphate
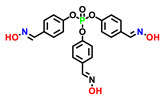
	TFP	tri(2-furyl) phosphoramide
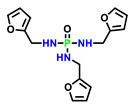
	TMCTP	Tri-Maleimide End-Capped Cyclotriphosphazene
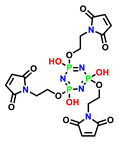
	BAPPO	bis(4-amino phenoxy)phenyl phosphine oxide
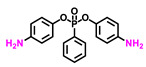
	BSPB	Benzoguanamine spirocyclic pentaerythritol bisphosphonate
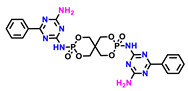
	OH_2_-N_2_P_4_	(bis((bis((l3-oxidaneylidyne)methyl)phosphoryl)methyl)amino)methyl 4-(2-(3-((bis((bis((l3-oxidaneylidyne)methyl)phosphoryl)methyl)glycyl)oxy)-2-hydroxypropoxy)ethoxy)-3-hydroxybutyrate
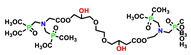
	JZP	2-(((diethoxyphosphoryl)methyl)(2-hydroxyethyl)amino)ethyl (6-hydroxyphenyl) adipate
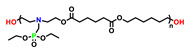
	PNP	18-(bis(hydroxymethyl)amino)-10-hydroxy-18-oxooctadecan-9-yl dipentyl phosphate
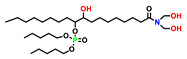
	FRD	10-(1,4-dicarboxyl)-9,10-dihydro-9-oxa-10-phosphaphenanthrene- 10-oxide-diethyl bis(2-hydroxyethyl) aminomethylphosphonate
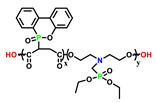
	DOPO-DAM	9,10-dihydro-9-oxa-10- [N, N-bis-(2- hydroxyethylamino-methyl)]-10-phosphaphenanthrene-10- oxide
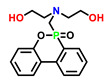
	PHID	4-DOPO-((3-hydroxypropyl) imino) methyl) phenol
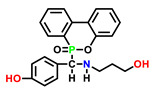
	DOPD-HAMB	4-DOPO-(((4-hydroxyphenyl)amino)methyl)benzene- 1,3-diol
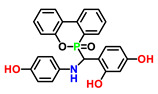
	P-S	phosphonate diol II
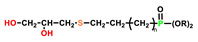
	P-Si(PHID+Si-OH)	4-DOPO-((3-hydroxypropyl) imino) methyl) phenol Hydroxy silicone oil
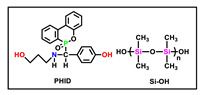
	P-N-S	vanillin-based polyol with flame-retardant group
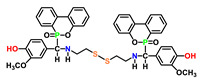
	HAMPP	2-((Bis(2-hydroxyethyl) amino) methyl)-5,5-dimethyl-1,3,2-dioxaphosphinane 2-oxide
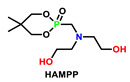
	PPA-PO-polyol	2-hydroxypropyl isobutyl phenylphosphonate
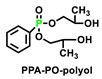
	PDEO	ethane-1,2-diyl bis(2-hydroxyethyl) bis(phenylphosphonate)
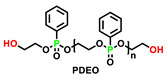
	DMOP	bis(2-((2-hydroxyethyl)amino)ethyl) methylphosphonate
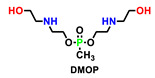
	aminophosphonate polyether polyol	aminophosphonate polyether polyol
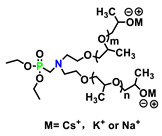
-	PEG	Polyethylene Glycol
-	PPG	Polypropylene Glycol
-	PTMEG	Polytetramethylene ether Glycol
-	TDI	Toluene di-isocyanate
-	MDI	Methylenediphenyl di-isocyanate
-	HDI	Hexamethylene di-isocyanate
-	IPDI	Isophorone di-isocyanate
-	HMDI	4,4′-diisocyanate dicyclohexylmethane
